# A fast navigator (fastNAV) for prospective respiratory motion correction in first‐pass myocardial perfusion imaging

**DOI:** 10.1002/mrm.28617

**Published:** 2020-12-03

**Authors:** Ronald Mooiweer, Radhouene Neji, Sarah McElroy, Muhummad Sohaib Nazir, Reza Razavi, Amedeo Chiribiri, Sébastien Roujol

**Affiliations:** ^1^ School of Biomedical Engineering and Imaging Sciences, Faculty of Life Sciences and Medicine King’s College London London United Kingdom; ^2^ MR Research Collaborations Siemens Healthcare Limited Frimley United Kingdom

**Keywords:** myocardial perfusion, navigator, prospective motion correction, respiratory motion correction, slice tracking, subject‐specific tracking factor

## Abstract

**Purpose:**

To develop and evaluate a fast respiratory navigator (fastNAV) for cardiac MR perfusion imaging with subject‐specific prospective slice tracking.

**Methods:**

A fastNAV was developed for dynamic contrast‐enhanced cardiac MR perfusion imaging by combining spatially nonselective saturation with slice‐selective tip‐up and slice‐selective excitation pulses. The excitation slice was angulated from the tip‐up slice in the transverse plane to overlap only in the right hemidiaphragm for suppression of signal outside the right hemidiaphragm. A calibration scan was developed to enable the estimation of subject‐specific tracking factors. Perfusion imaging using subject‐specific fastNAV‐based slice tracking was then compared to a conventional sequence (ie, without slice tracking) in 10 patients under free‐breathing conditions. Respiratory motion in perfusion images was quantitatively assessed by measuring the average overlap of the left ventricle across images (avDice, 0:no overlap/1:perfect overlap) and the average displacement of the center of mass of the left ventricle (avCoM). Image quality was subjectively assessed using a 4‐point scoring system (1: poor, 4: excellent).

**Results:**

The fastNAV calibration was successfully performed in all subjects (average tracking factor of 0.46 ± 0.13, R = 0.94 ± 0.03). Prospective motion correction using fastNAV led to higher avDice (0.94 ± 0.02 vs. 0.90 ± 0.03, *P* < .001) and reduced avCoM (4.03 ± 0.84 vs. 5.22 ± 1.22, *P* < .001). There were no statistically significant differences between the 2 sequences in terms of image quality (both sequences: median = 3 and interquartile range = 3‐4, *P* = 1).

**Conclusion:**

fastNAV enables fast and robust right hemidiaphragm motion tracking in a perfusion sequence. In combination with subject‐specific slice tracking, fastNAV reduces the effect of respiratory motion during free‐breathing cardiac MR perfusion imaging.

## 1 INTRODUCTION

First‐pass cardiac MR (CMR) perfusion imaging is widely used as part of the standard cardiac MR routine for patients with suspected coronary artery disease and is recommended for ischemia assessment in current guidelines in the United States and Europe.^1^, ^2^ In this technique, saturation recovery T_1_‐weighted images are dynamically acquired during the first‐pass of a T_1_‐shortening contrast agent,^3^ allowing perfusion defects to be identified as areas of reduced signal intensities.

CMR perfusion images are typically acquired during a prolonged breath hold aimed at covering the first pass of the contrast agent in the left ventricle (LV).^3^ However, breath holding may not always be an ideal solution due to patient‐related factors (such as inability to hold breath for a sufficient period) or due to technical factors (eg,incorrect timing of contrast administration). In addition, motion drift may be seen with prolonged breath holds.^4^ Alternatively, images can be acquired under free‐breathing conditions. In this case, respiratory motion manifests as in‐ and through‐plane variations of the imaged myocardium over time. Retrospective respiratory motion correction using image registration algorithms are often applied to reconstructed perfusion images.^5^ However, these techniques cannot compensate for through‐plane motion in 2D acquisitions and therefore also do not facilitate advanced acceleration/reconstruction techniques that exploit temporal information.^6^, ^7^, ^8^, ^9^, ^10^, ^11^, ^12^ Prospective respiratory motion correction can reduce through‐plane motion effects and improve the robustness of retrospective motion correction.^13^ Such correction could also be of benefit to visual perfusion analysis (by improving the consistency of imaged anatomy), quantification of perfusion (which requires temporal alignment of the myocardium to enable pixel‐wise fitting),^14^ and perfusion imaging during exercise stress when large breathing motion is typically observed.^15^ A minimal temporal footprint is also desirable for any prospective motion correction technique to maximize the time available for data sampling of the perfusion images.

The combination of a respiratory navigator (NAV)^16^, ^17^ and real‐time slice tracking has been proposed to prospectively reduce the amount of through‐plane respiratory motion in CMR images.^18^, ^19^ A NAV is commonly positioned on the dome of the right hemidiaphragm (RHD) to monitor the diaphragmatic motion that is strongly correlated to cardiac motion. Commonly in CMR prospective slice tracking, a fixed tracking factor of 0.6 is used to relate RHD motion to cardiac motion.^18^, ^19^ However, further reduction of the respiratory motion impact has been achieved when a subject‐specific tracking factor was used,^20^, ^21^ including for dynamic 2D imaging under free‐breathing conditions.

For CMR perfusion imaging, NAV‐based slice tracking was initially implemented by positioning a RHD NAV immediately before each saturation pulse.^13^ However, NAV signal quality was not always sufficient, potentially due to the repeated application of nonselective saturation pulses, and varying saturation recovery times in between heart beats could lead to fluctuating NAV signals. To reduce the influence of the prior magnetization history, a RHD NAV has been combined with spatially selective saturation pulses prescribed over the heart only; however, such pulses are often associated with reduced saturation efficiency.^22^, ^23^ Alternatively, Basha et al proposed to restore the RHD magnetization locally after the saturation pulse, followed by a standard RHD NAV acquisition.^15^ Using local magnetization restoration allows the NAV to be temporally close to image acquisition, which has been shown to improve the accuracy of slice tracking when compared to NAV positioned further away from the imaging,^24^ that is, before the saturation pulse. However, both NAV restore and acquisition used 2D‐selective excitation, resulting in prolonged acquisition time per saturation recovery image.

In this work, a fast RHD navigator (fastNAV) using slice‐selective tip‐up and excitation pulses is proposed to enable rapid tracking of the RHD motion in a perfusion sequence. Slice‐selective pulses can have a small temporal footprint, whereas angulation of these slices with respect to each other was used to localize the navigator signal to the RHD in the transverse plane. By applying the tip‐up pulse immediately following the saturation pulse and exciting the fastNAV with a low flip angle, signal variations were minimized. To enable the estimation of a subject‐specific tracking factor for fastNAV, a calibration scan was also developed. Perfusion imaging using subject‐specific fastNAV‐based slice‐tracking prospective motion correction was then compared to a conventional perfusion sequence in patients under free‐breathing conditions.

## 2 METHODS

### 2.1 Fast perfusion navigator

**FIGURE 1:**
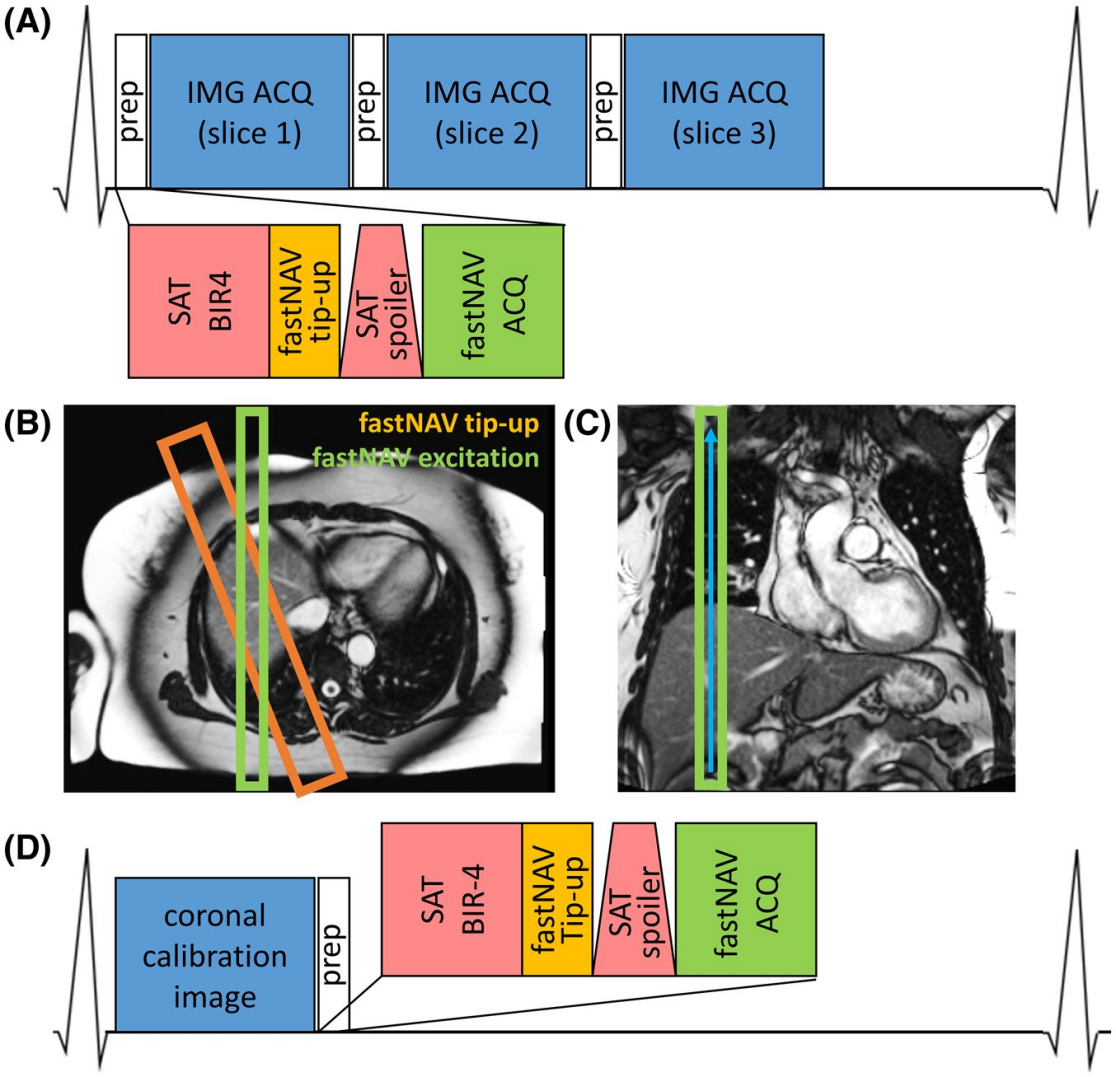
(A) Pulse sequence diagram of cardiac‐triggered CMR perfusion imaging with fastNAV. (B) Geometry of slice‐selective fastNAV pulses overlaid on axial localizer image. The orange rectangle marks the fastNAV tip‐up slice at twice the width of the fastNAV excitation slice (green rectangle). (C) Coronal localizer image showing fastNAV’s frequency encoding direction as blue arrow. (D) Pulse sequence diagram of the fastNAV calibration scan, where fastNAV is acquired after a coronal image of the LV per heartbeat. CMR, cardiac MR; fast NAV, fast navigator; fastNAV ACQ, acquisition of fastNAV using slice‐selective excitation (15**°**) and frequency‐encoded gradient echo; fastNAV tip‐up, slice‐selective −90° pulse; IMG ACQ, perfusion image acquisition using balanced steady‐state free precession; LV, left ventricle; prep, preparatory pulses for saturation recovery imaging including fastNAV; SAT BIR‐4, saturation with nonselective adiabatic BIR‐490° pulse; SAT spoiler, gradient spoiler to dephase remaining transverse magnetization after saturation pulse

The diagram of the proposed fastNAV prototype pulse sequence is shown in Figure 1A. The navigator signal is obtained by combining spatially nonselective saturation (necessary for perfusion imaging) with slice‐selective tip‐up and slice‐selective excitation pulses. To this end, a BIR‐4 90° adiabatic saturation pulse module^25^, ^26^ was modified by adding a slice‐selective tip‐up pulse (−90° flip angle, 40 mm thickness) between the adiabatic saturation pulse and the spoiler gradient. This slice‐selective tip‐up pulse restores the longitudinal magnetization along a section in the superoinferior plane (Figure 1B). The navigator signal is then obtained from an additional slice‐selective low flip angle excitation (15° flip angle, 20 mm thickness) in a superoinferior plane (sagittal slice, Figure 1B,C), angulated from the tip‐up slice in the transverse plane. The signal thus comes from the overlap area between these 2 slice‐selective pulses, prescribed to intersect the dome of the RHD. The gradient‐recalled navigator signal is (frequency) encoded in the superoinferior direction only (256 mm FOV, 0.5 mm resolution) (Figure 1C). The pulse structure of fastNAV resulted in a 9.5 ms contribution to the duration of each saturation recovery block (1.8 ms for tip‐up and 7.7 ms for fastNAV acquisition). To allow for navigator signal processing and feedback to be performed in real time, a 5 ms delay is added after the fastNAV acquisition, bringing the total additional scan time to 15 ms per navigator.

### 2.2 Prospective motion‐corrected perfusion imaging

The proposed perfusion sequence acquires 3 short axis slices per heartbeat, with prospective fastNAV‐based slice tracking applied to each slice (Figure 1A). The RHD displacement is extracted from the fastNAV signal with a conventional cross‐correlation analysis^16^ that is used by the vendor for conventional navigator‐based motion estimation. The displacement is then used for prospective real‐time adjustment of the imaging plane location in the superoinferior direction. A tracking factor is applied to account for the difference between the RHD displacement and the heart displacement along the superoinferior axis, as commonly performed in CMR.

### 2.3 Subject‐specific tracking factor calibration

In order to calibrate subject‐specific tracking factors, a sequence was designed to allow the near‐simultaneous measurement of fastNAV signal and LV displacement in a coronal image (Figure 1D). The calibration sequence is based on the electrocardiogram‐triggered perfusion sequence, as described in the previous section with 2 modifications. First, only 1 coronal image was acquired per heartbeat. Second, the order of the pre‐pulses and imaging was exchanged to provide higher SNR in the image while still acquiring a similar fastNAV signal as observed in the perfusion sequence (based on saturation, tip‐up, and excitation).

The fastNAV‐based RHD displacement values and coronal DICOM images were exported offline to estimate a subject‐specific tracking factor using a custom‐based software (MedIACare^27^) in MATLAB (The MathWorks, Natick, MA). A region of interest was drawn around the LV in the coronal image, for which the motion was determined using a rigid motion estimation algorithm (objective function: intercorrelation coefficient, optimization strategy: fixed‐step gradient descent) adapted from Ref. 28. After removal of their mean values, the slope between the 2 motion traces was found by least‐squares‐fitting a single parameter (slope), which was used as the subject‐specific tracking factor. The correlation coefficient (R) was also calculated and reported. Once the data were transferred offline to a laptop, calibration was typically done in 1 min. Of this calibration time, about 3 s were needed for calculations; the rest was needed for user interaction with the software.

### 2.4 In vivo evaluation

Ten patients (7 male, 3 female, mean age 46.9 ± 17.5 years) were included in this study. These patients were referred for an outpatient elective CMR study on clinical grounds, and their indications for the study included suspected cardiomyopathy (7) and suspected myocarditis (3). This study was approved by the National Research Ethics Service (15/NS/0030), and written informed consent was obtained from all patients for the scan and for inclusion in this study. Imaging was performed at 1.5 T (Magnetom Aera, Siemens Healthcare, Erlangen, Germany) using a 32‐channel spine array and an 18‐channel body array coil.

Each subject underwent a fastNAV calibration scan (Figure 1D) prior to contrast injection for estimation of a subject‐specific tracking factor, as described in the previous section. Imaging parameters were: TR/TE: 2.42/1.01 ms, FA: 50°, FOV: 400 × 400 mm^2^, slice number: 1, slice thickness: 10 mm, matric size: 192 × 192, voxel size: 2.1 × 2.1 mm^2^, temporal GRAPPA acceleration factor: 3, bandwidth: 1085 Hz/pixel, and dynamics: 30.

Two rest dynamic contrast‐enhanced perfusion protocols were then performed within the same CMR examination using 1) the proposed prospective motion correction approach, and 2) a conventional approach without motion correction. A dose of 0.075 mmol/kg gadobutrol (Gadovist, Bayer, Berlin, Germany) was injected at a rate of 4mL/s, followed by a 25 mL flush of normal saline by a power injector (Spectris Solaris EP, Medrad Inc., Warrendale, PA) for each perfusion protocol. To allow for contrast washout, the second perfusion protocol was performed 10 min after the first injection. The order of the 2 perfusion protocols was randomized across subjects. A BIR‐4 adiabatic saturation pulse was used in all cases, with no additional delay time between the fastNAV and the image acquisition to maximize the time available for data sampling of the perfusion image. Both perfusion acquisitions used an electrocardiogram‐triggered balanced steady‐state free precession saturation recovery sequence prescribed in the short axis orientation with the following parameters: TR/TE: 2.38/1.04 ms, FA: 50°, FOV: 360 × 270 mm^2^, slice number: 3, slice thickness: 8 mm, matrix size: 192 × 144, voxel size: 1.9 × 1.9 mm^2^, partial Fourier factor: 3/4, GRAPPA acceleration factor: 2 (24 integrated reference lines), bandwidth: 1085 Hz/pixel, total acquisition duration per heartbeat: 603.9 ms, effective saturation recovery time: 133 ms, and dynamics: 60. The FOV was adjusted according to patient size. Both sequences were planned on the systolic phase of 2‐, 3‐, and 4‐chamber cine images to ensure coverage of the base, mid‐, and apical slices. The patients were instructed to breathe normally for all scans (calibration scan and 2 perfusion scans) in this study.

### 2.5 Quantitative analysis

Although RHD NAV is expected to jointly reduce through‐plane and in‐plane motion, only in‐plane motion can confidently be assessed in 2D short axis perfusion images. To quantify any potential benefit of the proposed prospective motion correction, the temporal alignment of the LV in dynamic images was assessed by measuring the average Dice similarity coefficient^29^ of the LV over dynamics (avDice), as well as the average displacement of the LV center of mass location (avCoM). These metrics were computed offline using MedIACare^27^ in MATLAB (The MathWorks, Natick, MA) as follows. The epicardial contour of the LV myocardium was first manually delineated for each dynamic, starting from contrast arrival in the LV myocardium until the end of the time series. Dynamic images acquired prior to contrast arrival in the LV were discarded from this analysis because delineation of the LV epicardial contours is difficult to achieve in those images due to the lack of contrast. The avDice was computed as the average of the Dice similarity coefficient of the LV calculated for all possible pairs of dynamic images (including all nonconsecutive dynamic images). Similarly, avCoM was measured as the average displacement of the LV center of mass between all possible pairs of dynamic images. This analysis was performed independently for each slice of each dataset.

### 2.6 Qualitative analysis

To investigate whether fastNAV may have introduced additional image artefacts, subjective assessment of image quality was performed in consensus by 2 expert readers (s.n. and a.c.) with more than 5 and 10 years of CMR experience, respectively. The readers were blinded from both patient information and acquisition scheme. Images were exported in the DICOM format and visualized using OsiriX (Pixmeo SARL, Geneva, Switzerland). Image quality was assessed for each dataset using a 4‐point scoring system (4 = excellent, 3 = minor artefact but not limiting diagnosis, 2 = major artefact but not limiting diagnosis, 1 = poor image quality and nondiagnostic).

### 2.7 Statistical analysis

All results of continuous variables are expressed as mean ± SD, whereas results concerning nonparametric variables are presented as median value and interquartile range. Normality of distribution of continuous variables (avCoM and avDice) was assessed using the Shapiro‐Wilk test. Continuous variables were compared between the 2 sequences using paired *t* tests (for normally distributed variables) or Wilcoxon signed‐ranks test (for variables not normally distributed). Qualitative image quality scores were compared between the 2 sequences using the Wilcoxon signed‐ranks test. All statistical tests were 2‐tailed, and *P* values < .05 were considered significant.

## 3 RESULTS

### 3.1 Subject‐specific tracking factor calibration

**FIGURE 2:**
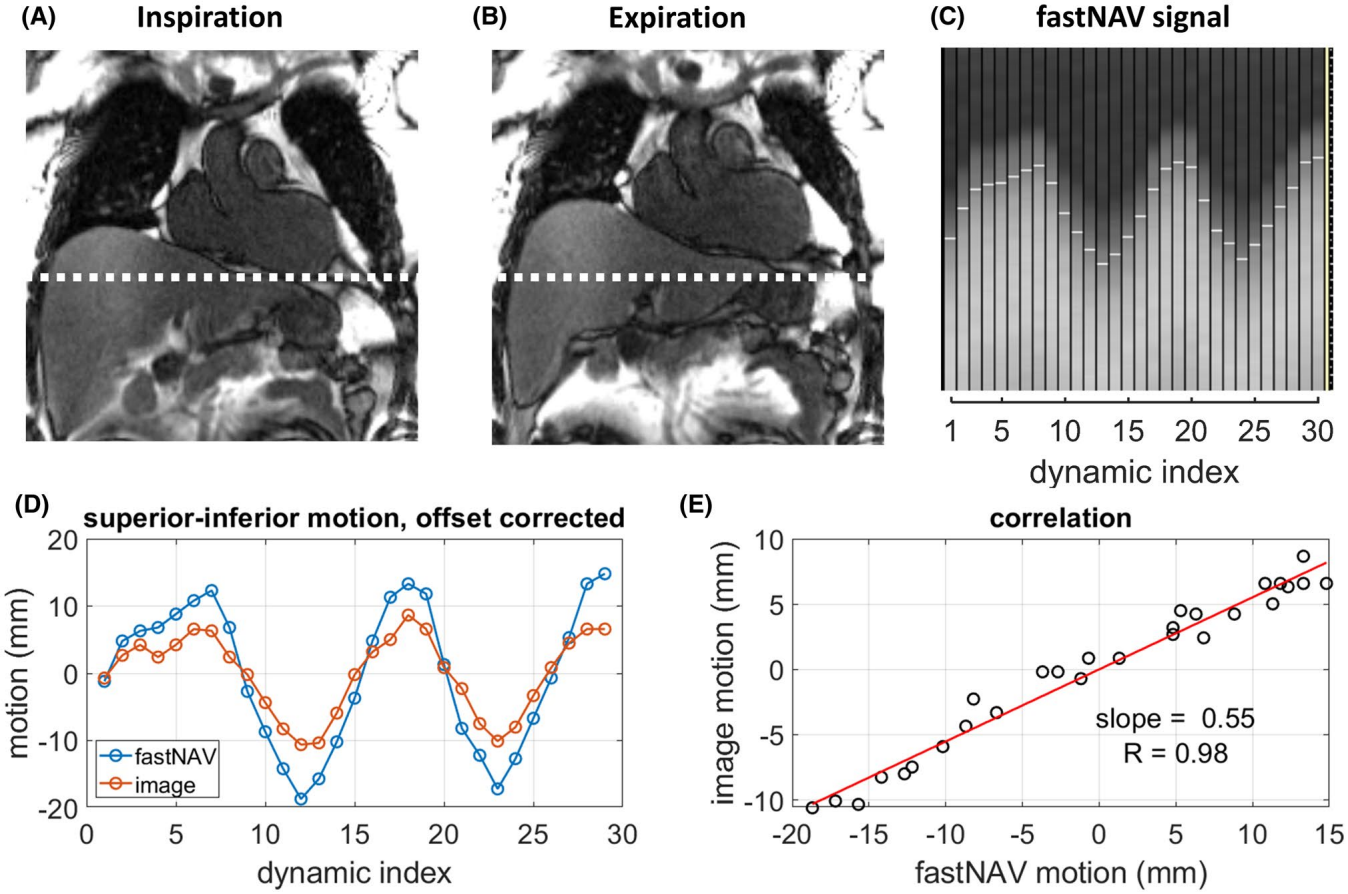
Calibration of the tracking factor with fastNAV. (A&B) Examples of coronal calibration images in inspiration ([A] dynamic index 13) and expiration ([B] dynamic index 18) that are used to detect LV motion. A dotted white line marks the inferior edge of the LV in inspiration to highlight the difference with expiration. After a region of interest (not shown) was manually drawn around the LV in 1 of the images, image registration was used to determine the motion of the LV across the time series. (C) Example of fastNAV signal for which white horizontal line segments mark the detected positions, which wereexported for tracking factor calibration. (D) Superoinferior fastNAV‐based RHD motion and imaged‐based LV motion after subtraction of their mean values. (E) Correlation between the fastNAV‐based and image‐based motion traces. The slope was found using least‐squares fitting and was used as subject‐specific tracking factor. Data shown is subject 3. RHD, right hemidiaphragm

Examples of the fastNAV signal and images acquired during a calibration scan in 1 patient are given in Figure 2. The fastNAV signal enables clear visualization of the lung/liver interface, displays a periodicity as expected from the breathing activity, and appears of consistent quality over time.

The fastNAV calibration was successfully performed in all subjects and is summarized in Table 1. Overall, the subject‐specific tracking factor was 0.46 ± 0.13 (minimum = 0.18, maximum = 0.68) with an R value of 0.94 ± 0.03 (minimum = 0.90, maximum = 0.98), indicative of a robust relationship between measured fastNAV and LV motion.

**TABLE 1:** Subject‐specific tracking factors

Subject	Tracking factor	R
1	0.40	0.97
2	0.40	0.93
3	0.55	0.98
4	0.40	0.94
5	0.48	0.90
6	0.42	0.90
7	0.64	0.95
8	0.34	0.96
9	0.28	0.92
10	0.68	0.94
Mean	0.46	0.94
SD	0.13	0.03
Minimum	0.28	0.90
Maximum	0.68	0.98

Tracking factor and R between fastNAV and LV motion for all subjects, followed by their respective mean value, SD, and minimum and maximum values.

LV, left ventricle; R, correlation coefficient.

### 3.2 Prospective motion‐corrected perfusion imaging

**FIGURE 3:**
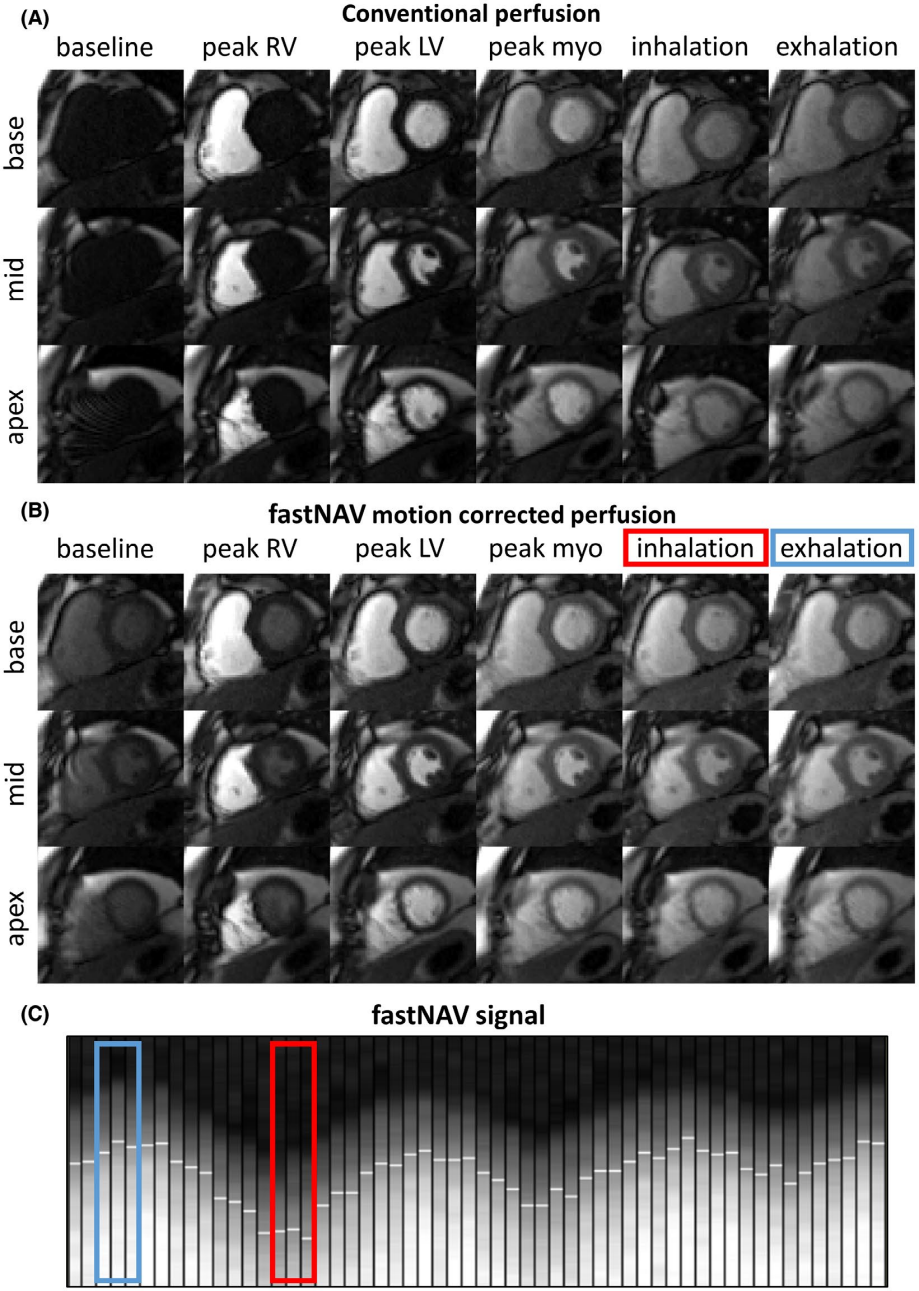
Examples of first‐pass perfusion images acquired in 1 subject (subject 3) under free‐breathing conditions using the conventional and proposed fastNAV approach. Full series are available as video in Supporting Information Videos S1 and S2. Representative dynamics acquired near end‐inspiration and end‐expiration are shown in the last 2 columns. (A) Conventional perfusion without motion correction. Images in end‐inspiration and end‐expiration were selected visually based on the heart’s vertical position. (B) Perfusion sequence with fastNAV motion correction. Images in end‐inspiration and end‐expiration were chosen based on the navigator trace. (C) Segment of the fastNAV signal trace where white horizontal line segments mark the detected navigator positions. The colored rectangles in B and C mark the navigator signal for the images in end‐inspiration (red) and end‐expiration (blue). Substantial motion can be observed between end‐inspiration and end‐expiration images acquired using the conventional sequence. The amount of motion between end‐inspiration and end‐expiration images was substantially reduced using the proposed fastNAV approach. LV, left ventricle; myo, myocardium; RV, right ventricle

Figure 3 shows examples of the perfusion images acquired in 1 subject (subject 3), together with a section of the corresponding fastNAV signal. The fastNAV signal quality is consistent with the signal quality observed during the calibration phase. Videos of the corresponding perfusion images are shown using the conventional perfusion acquired during the first contrast injection (Supporting Information Video S1) and the fastNAV perfusion acquired during the second contrast injection (Supporting Information Video S2). Videos of another case (subject 5) in which the order of the 2 perfusion scans was reversed are also shown for both conventional perfusion (Supporting Information Video S3) and fastNAV perfusion (Supporting Information Video S4). FastNAV‐based prospective slice tracking resulted in apparent reduction of residual in‐plane motion when compared to the conventional sequence. Both scans led to similar apparent image quality in all examples.

**FIGURE 4:**
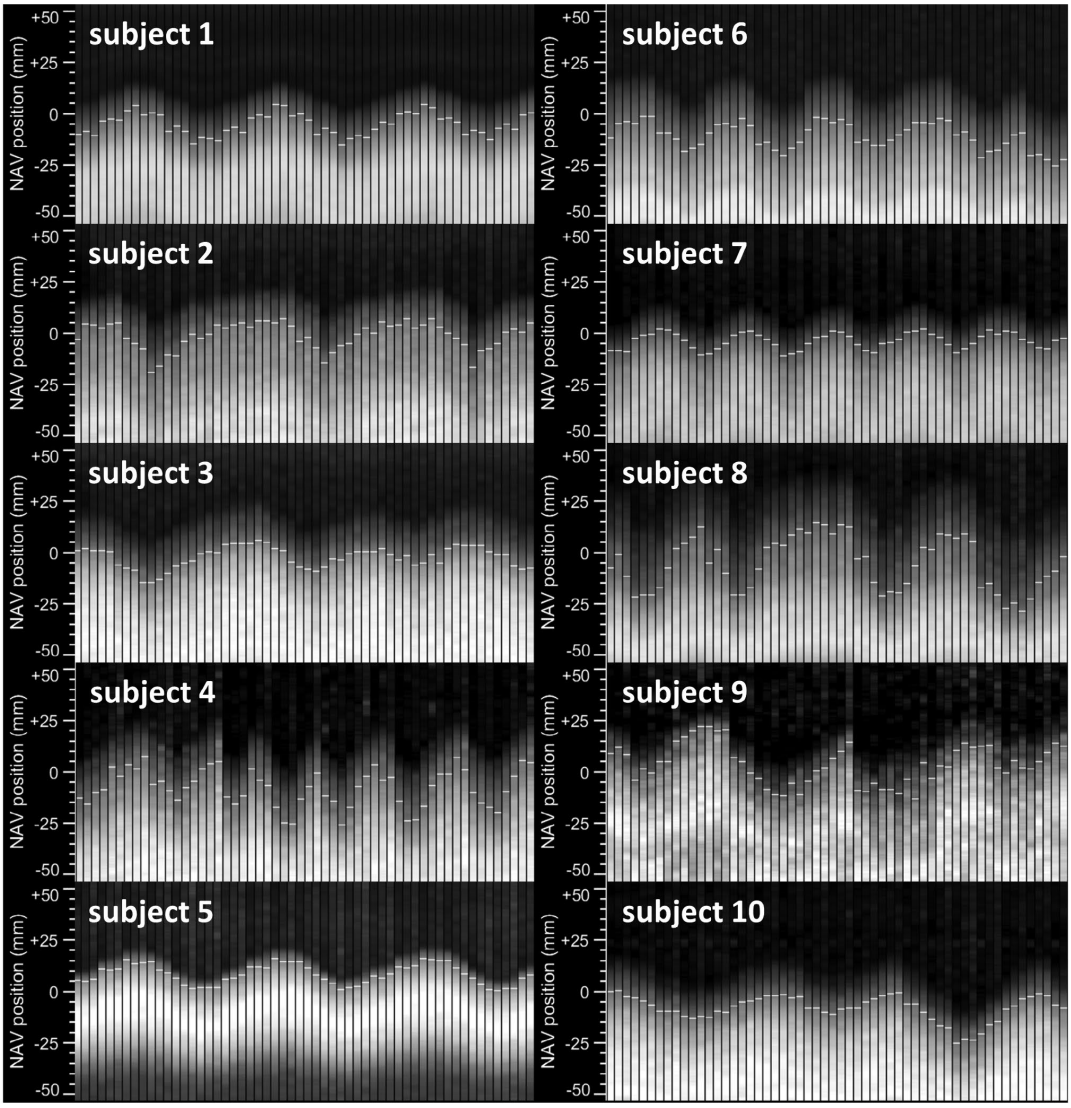
Signal examples of fastNAV acquired during perfusion imaging with fastNAV‐based motion correction for all subjects. Consecutive fastNAV signals acquired over the first 19 heartbeats of each dynamic acquisition (3 fastNAV signals per heartbeat;ie, 1 per slice) are shown for each subject. The fastNAV signal had high SNR and was consistent over time. The visual displacements observed in the fastNAV signal traces were successfully tracked (white horizontal marks)

For all 10 subjects, fastNAV signals from the first 19 dynamics from the start of the perfusion imaging are shown in Figure 4. The signals appear of high SNR and consistent through time, allowing the tracking of RHD displacement. In all subjects, the detected RHD displacement varies through time in a manner that is expected during free‐breathing. In subject 9, a sharp drop of the RHD level can be seen halfway in the panel, possibly due to a rapid exhalation.

**FIGURE 5:**
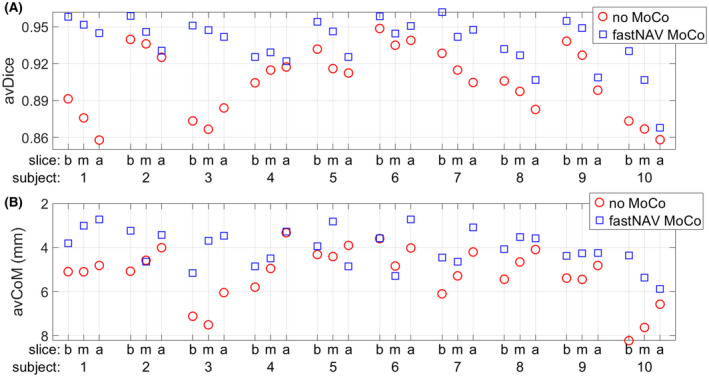
Quantitative motion analysis of perfusion images over all subjects using the conventional sequence (no MoCo, red circles) and the fastNAV prospective motion‐corrected sequence (fastNAV MoCo, blue squares). (A) Average overlap of the LV across images (avDice). (B) Average displacement of the LV center of mass (avCoM), with inverted *y*‐axis direction to aid the comparison with avDice (ie, a high position in the graph indicates little motion). All results are shown per subject and per slice (b/m/a). The proposed fastNAV led to higher avDice (0.94 ± 0.02 vs. 0.91 ± 0.03, *P* < .001) and reduced avCoM (4.03 ± 0.84 vs. 5.22 ± 1.22, *P* < .001) when compared to the conventional approach. a, apex; b, base; m, mid; MoCo; motion correction

Both fastNAV and conventional perfusion sequences were successfully acquired in all patients. Figure 5 shows avDice and avCoM measured over all slices and all subjects. In the majority of cases, images with fastNAV consistently display improved motion metrics. In subjects 9 and 10, the avDice value of the apical slice improved less compared to the base and mid slices. Videos of subject 10 (where the effect is most pronounced) are also provided (Supporting Information Videos S5 and S6, conventional and fastNAV perfusion, respectively). Although substantial reduction of motion can be observed in all slices, the apical slice depicts pronounced local nonrigid deformation of the myocardium in both sequences, to which the lower avDice score might be attributed. Over all subjects, the fastNAV‐enabled prospective motion correction led to higher avDice in comparison to the conventional sequence (0.94 ± 0.02 vs. 0.91 ± 0.03, *P* < .001) and reduced avCoM (4.03 ± 0.84 vs. 5.22 ± 1.22, *P* < .001).

All presented slices were scored either as “excellent” or “minor artefact but not limiting diagnosis.” Overall, there was no statistically significant difference between the 2 methods in terms of image quality (median = 3 and interquartile range = 3‐4 for both sequences, *P* = 1).

## 4 DISCUSSION

In this study, fastNAV has been developed for prospective motion correction of dynamic contrast‐enhanced perfusion imaging and was evaluated in patients undergoing clinical CMR. Subject‐specific slice tracking factors were calculated successfully for each subject. The use of fastNAV enabled robust RHD motion estimation during perfusion scans and substantially reduced the effect of respiratory motion in the perfusion images without affecting the image quality.

The fastNAV added only 15 ms to each saturation recovery block (of which 5 ms feedback time), which was shorter than previously reported navigators used for CMR perfusion imaging.^13^, ^30^, ^31^ Therefore, fastNAV could be introduced with minimal impact on the sequence and imaging parameters, which is especially important for perfusion sequences with minimal delay between saturation and imaging acquisition. The prescription of fastNAV was similar to that of conventional commercially available cross‐pair NAV,^16^ which should aid in clinical acceptability. The average subject‐specific tracking factor across subjects was 0.46, whereas the commonly used factor for conventional RHD NAV^3^ is 0.6. The discrepancy might be due to the small sample size in this study. Nevertheless, if subject‐specific calibration is not available, fastNAV can also be used with a fixed tracking factor.

Slice tracking using fastNAV was shown to successfully reduce in‐plane motion. Because the slice location adjustment was in the superoinferior direction, the in‐plane and through‐plane motions for images acquired in the short axis orientation were geometrically linked, and it can be inferred that through‐plane motion was also reduced. Moreover, from studies with RHD NAV, it is already known that slice tracking reduces through‐plane motion.^13^, ^18^ Overall, fastNAV‐enabled prospective slice tracking resulted in substantial reduction of the global respiratory motion of the heart, but the presence of respiratory‐induced local nonrigid deformation as observed in the apical slice of a few patients could not be corrected. Because fastNAV enables reduction of through and in‐plane motion and retrospective image registration algorithms only allow in‐plane motion correction (including nonrigid in‐plane motion), the combination of both technique appears as a promising direction to achieve accurate 3D motion compensation and will be investigated in the future.

A 10 min delay was employed between the 2 perfusion scans, which was not sufficient to allow for the complete washout of the contrast agent from the myocardium. To minimize the effect this had on our measurements, the acquisition order of the 2 sequences was randomized across patients. We did not compare fastNAV to other prospective motion correction strategies because it is difficult to perform more than 2 perfusion protocols per subject in the same session. A conventional perfusion protocol without prospective motion correction was used as reference, which is the most common approach used in the clinic. Perfusion quantification was not performed in this study due to the high contrast agent dose and lack of suitable approach for sampling of the arterial input function. However, the accuracy of perfusion quantification has already been demonstrated to improve with the presence of prospective slice tracking (as reduced spatial variability of estimates) and accurate in‐plane motion correction.^13^

In this study, the calibration process relied on a linear model of the RHD motion and the superoinferior heart motion, as commonly used clinically.^3^ Although this approach enables correction of through‐plane motion in the superoinferior direction, which is the main component of the respiratory motion, the anteroposterior and right–left motion of the heart caused by respiration remains uncorrected. The use of advanced calibration techniques^21^, ^23^ could be combined with fastNAV to enable 3D prospective motion correction, possibly also taking into account nonlinear relationships between LV and RHD, including the hysteresis effect.

The fastNAV technique has been used with an adiabatic saturation pulse and is compatible with conventional nonselective saturation pulses. However, composite pulses have also been proposed to further improve the saturation efficiency.^32^ Integration of fastNAV into composite pulses would require further optimization because magnetization restoration would need to be implemented before each of the several spoiling gradients within the RF pulse train.

The feasibility of fastNAV was demonstrated during rest perfusion in a small number of patients with no perfusion defects, thereby opening the way for improved perfusion image acquisition and analysis in the clinic. Future larger studies will be needed to characterize the clinical benefit of fastNAV under stress conditions in patients with coronary artery diseases. The validity of the tracking factor estimated at rest and used during stress conditions remains to be demonstrated. It may also be possible to acquire the fastNAV calibration scan immediately before the stress perfusion scan to avoid such potential limitation, which would require an online and automatic calculation of the tracking factor. Other potential applications for fastNAV‐enabled prospective motion correction include: drift correction during prolonged breath‐hold perfusion protocols, correction of large respiratory motion occurring during exercise stress perfusion protocols, improvement of retrospective motion correction, improvement of reconstruction techniques exploiting temporal information, and possibly even reconstruction algorithms simultaneously solving image reconstruction and motion correction problems.^33^, ^34^, ^35^

## 5 CONCLUSION

The fastNAV enabled fast and robust RHD motion tracking in a CMR perfusion sequence. The use of fastNAV combined with real‐time slice tracking and a subject‐specific tracking factor reduced the effect of respiratory motion during free‐breathing dynamic contrast‐enhanced CMR perfusion imaging.

## CONFLICT OF INTEREST

Dr. Radhouene Neji is an employee of Siemens Healthcare.

## Supporting information


**VIDEO S1** Video of the entire dynamic perfusion imaging series acquired with the conventional non‐motion corrected sequence for subject #3. Base, mid, and apical images are displayed at 10 frames per second. The video shows the position of the myocardium moving due to respiratory motion

**VIDEO S2** Video of the entire dynamic perfusion imaging series acquired with the fastNAV‐enabled prospective motion corrected sequence for subject #3. Base, mid, and apical images are displayed at 10 frames per second. The video demonstrates that the position of the myocardium remains relatively fixed, with little residual motion

**VIDEO S3** Video of the entire dynamic perfusion imaging series acquired with the conventional non‐motion corrected sequence for subject #5. Base, mid, and apical images are displayed at 10 frames per second. The video shows the position of the myocardium moving due to respiratory motion

**VIDEO S4** Video of the entire dynamic perfusion imaging series acquired with the fastNAV‐enabled motion corrected sequence for subject #5. Base, mid, and apical images are displayed at 10 frames per second. The video demonstrates that the position of the myocardium remains relatively fixed, with little residual motion

**VIDEO S5** Video of the entire dynamic perfusion imaging series acquired with the conventional non‐motion corrected sequence for subject #10. Base, mid, and apical images are displayed at 10 frames per second. The video shows the position of the myocardium moving due to respiratory motion and non‐rigid deformation of the myocardium in the apical slice

**VIDEO S6** Video of the entire dynamic perfusion imaging series acquired with the fastNAV‐enabled motion corrected sequence for subject #10. Base, mid, and apical images are displayed at 10 frames per second. The video demonstrates that the position of the myocardium remains relatively fixed, with little residual motion but also non‐rigid deformation of the myocardium in the apical slice

## Data Availability

The data that support the findings of this study are available from the corresponding author (s.r.) upon reasonable request.
